# Higher comorbidities and early death in hospitalized African-American patients with Covid-19

**DOI:** 10.1186/s12879-021-05782-9

**Published:** 2021-01-18

**Authors:** Raavi Gupta, Raag Agrawal, Zaheer Bukhari, Absia Jabbar, Donghai Wang, John Diks, Mohamed Alshal, Dokpe Yvonne Emechebe, F. Charles Brunicardi, Jason M. Lazar, Robert Chamberlain, Aaliya Burza, M. A. Haseeb

**Affiliations:** 1 SUNY Downstate Medical Center, Departments of Pathology and Cell Biology, 450 Clarkson Ave. MSC #37, Brooklyn, NY 11203 USA; 2 Department of Pathology, SUNY Downstate Medical Center, Brooklyn, USA; 3 Department of Surgery, SUNY Downstate Medical Center, Brooklyn, USA; 4 Division of Cardiology, Department of Medicine, SUNY Downstate Medical Center, Brooklyn, USA; 5 Department of Anesthesiology, SUNY Downstate Medical Center, Brooklyn, USA; 6 Division of Pulmonary Medicine and Critical Care, Department of Medicine, SUNY Downstate Medical Center, Brooklyn, USA

**Keywords:** Health disparities, COVID-19, African-Americans, Dialysis, ACE inhibitors, Angiotensin II receptor blockers, Comorbidities, Chronic kidney disease

## Abstract

**Background:**

African-Americans/Blacks have suffered higher morbidity and mortality from COVID-19 than all other racial groups. This study aims to identify the causes of this health disparity, determine prognostic indicators, and assess efficacy of treatment interventions.

**Methods:**

We performed a retrospective cohort study of clinical features and laboratory data of COVID-19 patients admitted over a 52-day period at the height of the pandemic in the United States. This study was performed at an urban academic medical center in New York City, declared a COVID-only facility, serving a majority Black population.

**Results:**

Of the 1103 consecutive patients who tested positive for COVID-19, 529 required hospitalization and were included in the study. 88% of patients were Black; and a majority (52%) were 61–80 years old with a mean body mass index in the “obese” range. 98% had one or more comorbidities. Hypertension was the most common (79%) pre-existing condition followed by diabetes mellitus (56%) and chronic kidney disease (17%). Patients with chronic kidney disease who received hemodialysis were found to have lower mortality, than those who did not receive it, suggesting benefit from hemodialysis Age > 60 years and coronary artery disease were independent predictors of mortality in multivariate analysis. Cox proportional hazards modeling for time to death demonstrated a significantly high ratio for COPD/Asthma, and favorable effects on outcomes for pre-admission ACE inhibitors and ARBs. CRP (180, 283 mg/L), LDH (551, 638 U/L), glucose (182, 163 mg/dL), procalcitonin (1.03, 1.68 ng/mL), and neutrophil:lymphocyte ratio (8.3:10.0) were predictive of mortality on admission and at 48–96 h. Of the 529 inpatients 48% died, and one third of them died within the first 3 days of admission. 159/529patients received invasive mechanical ventilation, of which 86% died and of the remaining 370 patients, 30% died.

**Conclusions:**

COVID-19 patients in our predominantly Black neighborhood had higher in-hospital mortality, likely due to higher prevalence of comorbidities. Early dialysis and pre-admission intake of ACE inhibitors/ARBs improved patient outcomes. Early escalation of care based on comorbidities and key laboratory indicators is critical for improving outcomes in African-American patients.

**Supplementary Information:**

The online version contains supplementary material available at 10.1186/s12879-021-05782-9.

## Background

Coronavirus Disease 2019 (COVID-19), caused by infection with Severe Acute Respiratory Syndrome Coronavirus-2, has been declared by the World Health Organization to be a pandemic, with over seven million confirmed cases in the United States [1, 2]. New York State, including the New York City, became the epicenter of the epidemic in the United States, accounting for more than 23% of the total U.S. cases by the end of May, 2020 [2]. Such burden of disease is of particular concern since it disproportionately affects communities with considerable health disparities in New York City, where African-Americans and Latinos constitute as much as 53% of the population [3]. Our medical center is located in such a community in Brooklyn, New York.

The spectrum of COVID-19 presentation ranges from mild influenza-like illness to life-threatening severe respiratory disease requiring ventilatory support [3]. Comorbid conditions such as hypertension, diabetes mellitus, pulmonary and heart diseases, and demographic factors have been reported to influence outcomes [4–6]. However, the relative influence of each of these comorbidities in different patient populations and age strata has not been assessed, leading to variability in management and outcomes. Key decisions in patient management such as the choice of antibiotic, blood pressure goals, and perhaps most importantly, airway management strategies, have remained variable across or within hospitals.

National health statistics have documented extensive health disparities for Black COVID-19 patients. They suffer a three-fold greater infection rate, and a six-fold greater mortality rate than their white counterparts [7]. However, limited clinical and laboratory data of prognostic significance from Black COVID-19 patients are available [8]. A range of cultural, linguistic, and healthcare access barriers have prevented clinical investigation. Our hospital, located in New York City, serves a predominantly Black population, and being declared a COVID-only facility, we were able to maintain a standard quality-of-care across all COVID-19 patients.

Here we explore the clinical aspects of COVID-19 and its outcomes in Black patients. This study evaluated clinical signs and symptoms, laboratory indicators, and management strategies to develop a data-driven COVID-19 patient-care approach. Our findings provide an evidence-based resource for physicians to assess patient progress in the early days of hospitalization to direct patient management decisions.

## Methods

This study analyzed the electronic medical records of COVID-19 patients hospitalized at the State University of New York (SUNY), Downstate Medical Center, Brooklyn, New York. The hospital was designated a COVID-only facility by the State of New York as of March 4th, 2020, and provided ample equipment and supplies. The hospital is located in a majority Black neighborhood with high rates of poverty [9]. This study was approved by the SUNY Downstate Institutional Review Board [1587476–1].

COVID-19 diagnosis was based on clinical presentation and a positive real-time reverse transcriptase polymerase chain reaction (rtPCR) from a nasopharyngeal swab (Xpert Xpress SARS-CoV-2, Cepheid, Sunnyvale, CA). Of the 1103 patients who tested positive over a 52-day period (March 2nd – April 23rd), when the hospital was under peak caseload; 529, who met the following criteria were admitted and included in this study. Patients were admitted if deemed to be in respiratory distress (respiratory rate > 22 breaths/min and in need of supplemental oxygen to maintain oxygen saturation > 92%), were encephalopathic, or were judged sufficiently ill to require hospitalization. Patients were followed up for up to 7 months, thus we have been able to document an outcome (death or discharge) on all patients. COVID-19 positive pregnant patients who came for obstetrics related visit, and otherwise asymptomatic, were excluded.

Demographic factors, comorbidities, presenting clinical symptoms, and outcomes (discharge/death) were recorded for 529 patients. Complete medical history was available for 484 of these patients, however, 45 patients were too sick to respond or were in altered mental status at presentation and were excluded from analyses of co-morbidities. Laboratory data were recorded for 286 patients on admission or within 24 h of hospitalization, and at a second time point between 48 and 96 h post-admission. Pre-admission medications were recorded based on admission medication reconciliation by admitting physicians. Based on self-reported race/ethnicity, patients were grouped into Black and Others (White Hispanic/non-Hispanic and Asian). HIV-positive patients [with CD4 counts < 50% of the lower limit of the reference range (404–1612/μL)] and transplant recipients were categorized as “immunocompromised”. Chronic kidney disease (CKD) was defined as kidney damage and reduced glomerular filtration rate (GFR < 60 ml/min/1.73 m^2^) of more than 3 months [10]. We separated patients with kidney disease into 3 groups: 1) CKD without dialysis, defined as patients who were admitted with baseline CKD and did not receive dialysis during hospitalization; 2) CKD with dialysis, defined as patients with baseline CKD who started dialysis as inpatients because of worsened acute kidney injury; 3) ESRD, defined as patients who were on dialysis prior to admission and continued dialysis as per their routine schedule during hospitalization.

Patients were treated with hydroxychloroquine (200 mg twice a day, for 5 days) and azithromycin (250 mg once a day, for 5 days). All patients received standard venous thromboembolism prophylaxis with low-molecular weight heparin or direct oral anticoagulants based on their creatinine clearance rate. Patients with elevated D-dimer received a full dose anticoagulation regimen. Hypoxia, a sign of Acute Respiratory Distress Syndrome (ARDS), was monitored by a continuous pulse oximeter and with arterial blood gas measurements, and supplemental oxygen was provided as needed via noninvasive ventilation. Patients with worsening respiratory distress despite supportive care, as determined by declining pulse oximeter saturation, increasing respiratory rate, or worsening partial pressure of arterial oxygen/percentage of inspired oxygen ratio) were intubated and placed on mechanical ventilation. Patients who developed acute kidney injury (AKI) with oliguria (< 30 ml/hr. for > 12 h) unresponsive to diuretics or hemodynamic optimization, or decreased creatinine clearance (CrCl < 20 ml/min) received hemodialysis [11].

Computational analysis was conducted using R (ver. 3.6.3) [12]. Continuous variables are presented as median and interquartile range (IQR). Categorical variables such as gender or race are presented as number and percent of patients with 95% confidence intervals (CI). Percentages are expressed based on the available data for the subgroup relative to the total available data for that variable.

Parametric variables were evaluated through a Shapiro-Wilk test of normality with a significance cutoff of *P <* 0.01. Non-parametric variables were compared using Mann-Whitney rank sum test, with 95% CIs reported. Categorical variables were evaluated using the Fisher exact test, and odds ratios (OR) alongside 95% CIs are presented. All tests were two-tailed and statistical significance was defined as *P <* 0.05. No multiple testing correction was applied. A multivariate logistic regression analysis was performed on comorbidities and demographic factors for in-hospital mortality, and ORs with 95% CIs are presented. Cox proportional hazards analysis for time to death was conducted on comorbidities, demographic factors, and pre-admission medications [(angiotensin-converting enzyme (ACE) inhibitors and/or angiotensin II receptor blockers (ARBs)] and hazard ratios with 95% Cis are presented.

## Results

One thousand one hundred three patients were tested for COVID-19 over a 52-day period. After excluding 292 patients who tested negative and 282 who were treated as outpatients, 529 inpatients with positive test results and symptoms consistent with COVID-19 were included in this study, and were followed-up for up to 7 months.

### Demographic information

**Fig. 1:**
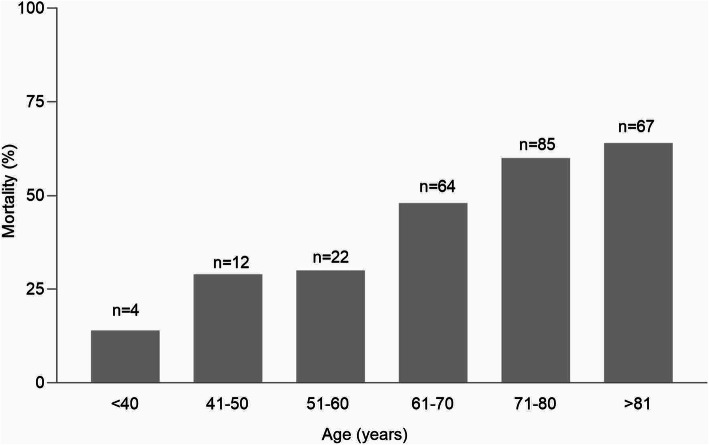
In-hospital mortality of COVID-19 patients in different age groups. The number of patients in each age-group are shown above the bars

The median patient age was 70 years (Table 1). A majority of patients were in the age range of 61–80 years (53%, 281/529) and a small minority were < 40 years old (6%, 28/529). In-hospital mortality rates correlated with patient age, with the highest mortality rate recorded for the > 80-year age group (64%, 67/104) (Fig. 1). 88% of the patients were Black (466/529) and the remaining 12% were Others. No difference in mortality rates were found between the two groups. Male-to-female ratio was 1.17:1, with a higher mortality rate for males (52%, 148/286). The mean BMI of patients was 30 kg/m^2^ (obese) and no correlation with mortality was found. A majority of patients (81%, 157/194) never smoked and, while not statistically significant, mortality rate increased with any history of smoking (Table 1).

**Table 1:** Demographic characteristics and outcomes of Covid-19 patients admitted for treatment. The number and percentage of patients for each variable are provided in columns “survivor” and “non-survivor”. The *P* values are based on comparisons between “survivor” and “non-survivor” patients. BMI, body-mass index; CI, confidence interval

Variable	Patients	Survivors	Non-survivors	Odds Ratio (95% CI)	***P*** value
Age - median	70	66	73	NA	< 0.001
*Age ranges*	*No./total no. (%)*	*no. (%)*	*no. (%)*		
+ 80 yr.	104/529 (20)	37 (36)	67 (64)	2.21 (1.39–3.57)	< 0.001
71–80 yr.	147/529 (28)	62 (44)	85 (60)	1.70 (1.11–2.53)	0.006
61–70 yr.	134/529 (25)	70 (52)	64 (48)	0.97 (0.64–1.47)	0.92
51–60 yr.	74/529 (14)	51 (70)	22 (30)	0.41 (0.23–0.72)	< 0.001
41–50 yr.	42/529 (8)	30 (71)	12 (29)	0.40 (0.18–0.84)	0.009
0–40 yr.	28/529 (5.7)	24 (86)	4 (14)	0.16 (0.04–0.48)	< 0.001
*Race/Ethnicity*	*no./total no. (%)*	*no. (%)*	*no. (%)*		
Black	466/529 (88)	244 (52)	222 (48)	0.77 (0.43–1.36)	0.41
Others	63/529 (12)	30 (48)	33 (52)	1.29 (0.73–2.30)	0.41
*Sex*	*no./total no. (%)*	*no. (%)*	*no. (%)*		
Male	286/529 (54)	138 (48)	148 (52)	1.37 (0.96–1.96)	0.08
Female	243/529 (46)	136 (56)	106 (44)	0.72 (0.50–1.04)	0.08
*BMI mean*	30	31	29	NA	0.40
*BMI*	*no./total no. (%)*	*no. (%)*	*no. (%)*		
< 29.9	133/238 (56)	46 (34)	87 (66)	1.25 (0.71–2.21)	0.41
> 30	105/238 (44)	42 (40)	63 (60)	0.79 (0.45–1.39)	0.41
*Smoking Status*	*no./total no. (%)*	*no. (%)*	*no. (%)*		
Non-smoker	161/200 (81)	82 (51)	79 (49)	0.74 (0.34–1.59)	0.47
Past/current smoker	39/200 (19)	17 (42)	22 (58)	1.34 (0.62–2.90)	0.47

### Presenting signs and symptoms, comorbidities, and pre-admission medication

Presenting patient complaints, grouped based on systemic symptoms, were fever (42%), respiratory (76%; cough, shortness of breath), gastrointestinal (21%; diarrhea, vomiting), and neurological (16%; altered mental status, seizure, unresponsiveness).

Comorbidities were present in 98% (517/529) of patients (Table 2). The most common comorbidities were hypertension (HT) (79%, 416/517) and diabetes mellitus (DM) (56%, 289/517), followed by chronic kidney disease (CKD (17%, 84/504)), (%,), hyperlipidemia (16%, 82/529), end stage renal disease (ESRD) (10%, 50/504), history of cancer (9%, 43/496), coronary artery disease (CAD) (8%, 42/529), chronic obstructive pulmonary disease (COPD) (7%, 36/481), and asthma (6%, 30/475). These comorbidities showed correlation with increased mortality except for HT. Autoimmune diseases (37/495) did not affect outcomes (Table 2). Patients with CKD on dialysis (2%, 11/504) showed lower mortality (*P =* 0.06) than counterparts with CKD without dialysis (14%, 73/504). Patients with ESRD (all on dialysis) showed a significantly higher survival in univariate analysis (*P* = 0.02) (Table 2). These results are notable considering patients with CKD and ESRD suffered higher mean number of comorbidities (mean 4.2) than other patients (mean 3.3, *P* < 0.001).

**Table 2:** Comorbidities among Covid-19 patients admitted for treatment. The number and percentage of patients for each variable are provided in columns “survivor” and “non-survivor”. The *P* values is are based on comparisons between “survivor” and “non-survivor” patients. CKD, chronic kidney disease; COPD, chronic obstructive pulmonary disease; ESRD, end-stage renal disease

Comorbidities	All Patients *no./total (%)*	Survivors *no. (%)*	Non-survivors *no. (%)*	Odds Ratio *(95% CI)*	***P*** value
Asthma	30/475 (6)	9 (30)	21 (70)	2.77 (1.18–7.04)	0.01
Autoimmune disease	37/495 (7)	22 (59)	15 (41)	0.71 (0.33–1.47)	0.39
History of cancer	43/496 (9)	14 (33)	29 (67)	2.39 (1.18–5.03)	0.010
COPD	36/481 (7)	16 (44)	20 (56)	1.48 (0.71–3.16)	0.297
Coronary Artery Disease	42/529 (8)	10 (24)	32 (76)	3.77 (1.76–8.81)	< 0.001
Congestive Heart Failure	25/529 (5)	16 (64)	9 (36)	0.59 (0.22–1.45)	0.22
CKD without dialysis	73/504 (14)	28 (38)	45 (62)	1.88 (1.11–3.27)	0.016
CKD with dialysis	11/504 (2)	9 (81)	2 (18)	0.23 (0.02–1.14)	0.06
ESRD on dialysis	50/504 (10)	34 (68)	16 (32)	0.47 (0.23–0.90)	0.02
Diabetes mellitus	289/517 (56)	139 (48)	150 (52)	1.48 (1.03–2.13)	0.03
Hyperlipidemia	82/529 (16)	34 (42)	48 (58)	1.63 (0.98–2.72)	0.05
Hypertension	416/517 (79)	212 (51)	204 (49)	1.35 (0.85–2.15)	0.184
Immune suppression	25/489 (5)	17 (68)	8 (32)	0.48 (0.17–1.21)	0.102
All patients ≥ 1 Comorbidities	517/529 (98)	271 (99)	246 (96)	–	–

**Fig. 2:**
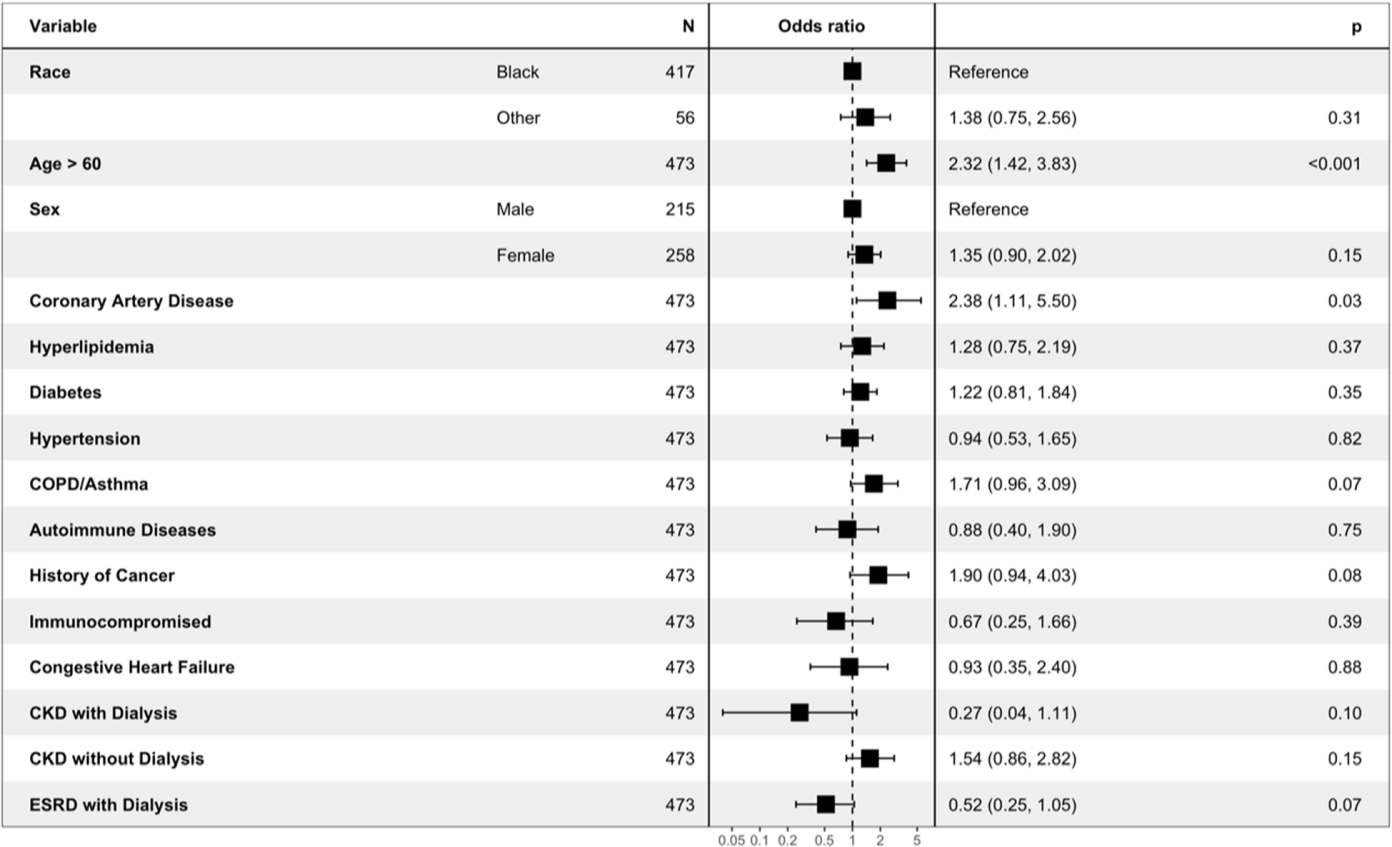
Multivariate logistic regression analysis of the demographic characteristics and comorbidities for mortality. The presented odds ratios have been adjusted for multiple testing. CKD, chronic kidney disease; COPD, chronic obstructive pulmonary disease; ESRD, end-stage renal disease

In multivariate analysis, age > 60 years and CAD were independent predictors of mortality. CKD patients who did not receive dialysis had a greater chance of death than those who were dialyzed (*P =* 0.15, OR, 1.54), and ESRD patients on dialysis had a lower risk of death (*P* = 0.07, OR, 0.52) (Fig. 2). Multivariate analysis (model 2) shows that patients who have CKD and/or ESRD as a comorbidity have a higher mortality, however, if dialysis is introduced as an intervention they have a significant survival advantage (*P* = 0.004) (Suppl. 1). Cox proportional hazards analysis for time to death showed that COPD/Asthma had a significantly higher hazards ratio for death (HR:1.79; CI: 1.20, 2.68; *P* = 0.005), and that pre-admission ACE inhibitors (20%, 29/142) and ARBs (25%, 35/142) had a beneficial effect (*P* = 0.013 and 0.036, respectively).

Complications during clinical course in 312 patients were acute hypoxic respiratory failure (37%), AKI (15%), cardiogenic shock (18%), neurological shock (5%), sepsis (4%), and diabetic ketoacidosis (3%).

### Laboratory data

At admission and at 48–96 h, leukocyte (8.6 K/μL, 10.6 K/μL) and neutrophil counts (7.3 K/μL, 8.9 K/μL) were higher (*P <* 0.001) and lymphocyte counts (0.8 K/μL) were lower at 48–96 h (*P =* 0.003) for non-survivors. The median neutrophil:lymphocyte ratio (NLR) was higher both at admission and at the second time point in patients who did not survive (8.3,10, *P* < 0.001*)*. Platelet and hemoglobin were marginally decreased but were not significantly different in survivors and non-survivors. Blood urea nitrogen (BUN) (33, 38 mg/dL), creatinine (1.7, 1.6 mg/dL), glucose (182, 163 mg/dL), alkaline phosphatase (66, 75 U/L), and aspartate aminotransferase (AST) (52, 64 μ/L) levels were higher in non-survivors at both time points (*P* < 0.001). Bilirubin and total protein were mildly increased in non-survivors, but were within their respective reference ranges. Albumin (3.4, 2.8 g/dL) was lower for non-survivors at both time points (*P* < 0.001). Lactate dehydrogenase (551, 638 U/L), C-reactive protein (180, 283 mg/L), and procalcitonin (1.03, 1.68 ng/mL) showed significantly higher serum levels at admission and at 48–96 h (*P* < 0.05) for non-survivors. D-dimer (3.0 mcg/mL, 7.5 times elevation), prothrombin time (PT) (17.2 s), and international normalized ratio (1.4 U) were increased in non-survivors at the second time point (*P* < 0.05). Activated partial thromboplastin time (aPTT) was not found to be different in the two groups (Table 3).

**Table 3:** Laboratory data of 286 inpatients at admission and at a secondary time point between 48 and 96 h of admission. Median and interquartile ranges are presented. The *P value* is calculated between patients who survived and did not survive. aPTT, activated partial thromboplastin time; Alk Phosphatase, alkaline phosphatase; ALT, alanine aminotransferase; AST, aspartate aminotransferase; BUN, blood urea nitrogen; CI, confidence interval; CRP, C-reactive protein; INR, international normalized ratio; LDH, lactate dehydrogenase; PT, prothrombin time

Laboratory values (*reference range*)	Time of determination (n)	Survivors	Non-survivors	95% CI	*P* value
		Median (Inter Quartile Range)			
*Hematologic parameters*					
Hemoglobin *(12–16 g/dL)*	At admission (286)	12.3 (11.0–14.0)	12.7 (11.3–14.3)	−0.9 - 0.1	0.13
	48–96 h (228)	12.0 (10.15–13.1)	11.7 (10.5–13.3)	−0.6 - 0.5	0.83
Leukocyte count *(3.5–10.8 K/μL)*	At admission (285)	7.2 (5.4–9.3)	8.6 (6.4–11.3)	−2.3 - -0.7	< 0.001
	48–96 h (228)	6.5 (5.1–9.4)	10.6 (7.8–14.3)	−4.9 - -2.7	< 0.001
Neutrophil Count *(1.7–7 K/*μL*)*	on admission (270)	5.8 (3.8–7.8)	7.3 (4.8–10.0)	− 2.3 - -0.8	< 0.001
	48–96 h (205)	5.4 (3.4–7.6)	8.9 (6.4–12.5)	−4.8, − 2.5	< 0.001
Lymphocyte count *(0.9–2.9 K/****μ****L)*	on admission (260)	0.9 (0.7–1.1)	0.8 (0.6–1.1)	−5.4e - -5, 0.2	0.04
	48–96 h(205)	1.0 (0.8–1.3)	0.8 (0.5–1.2)	0.1–0.3	0.002
Neutrophil Lymphocyte count (NLR)	on admission (260)	5.4 (3.7–8.1)	8.3 (5.3–13.7)	−3.8 - -1.4	< 0.001
	48–96 h (204)	4.7 (3.3–7.0)	10.0 (6.06–19.5)	−6.7 - -3.2	< 0.001
Eosinophil count *(0.0–0.8 K/****μ****L)*	on admission (255)	0.03 (0.01–0.07)	0.02 (0.01–0.04)	0.002–0.01	< 0.001
	48–96 h (203)	0.05 (0.02–0.1)	0.01 (0.01–0.04)	0.01–0.03	< 0.001
Platelet count *(130–400 K/****μ****L)*	on admission (283)	204 (158–266)	200 (147–260)	−11.0 - 28	0.40
	48–96 h (225)	229 (153–338)	194 (150–280)	− 5.9 - 53.9	0.11
*Blood Chemistry*					
Sodium *(136–145 mmol/L)*	on admission (286)	136 (133–138)	136 (132–141)	− 2.0 - 1.0	0.51
	48–96 h (237)	138 (136–140)	142 (137–147)	− 5.9 - -2.0	< 0.001
Potassium *(3.5–5.1 mmol/L)*	on admission (286)	4.2 (3.8–4.8)	4.4 (3.9–5.0)	− 0.4 - 4.9e-5	0.04
	48–96 h (235)	4.3 (4.0–4.6)	4.4 (3.9–5)	− 0.4 - 5.2e-5	0.05
Bicarbonate *(23.0–28.0 mmol/L)*	on admission (196)	25 (22–30)	22 (19–26)	1.0–4.9	0.001
	48–96 h (143)	24 (21–28)	21 (18–24)	1.6–5.0	<.0.001
Chloride *(98–107 mmol/L)*	on admission (285)	100 (94–103)	100 (96–105)	− 3.9 - 3.5e-5	0.09
	48–96 h (238)	102 (96–106)	107 (101–113)	− 8.0 - -3.0	< 0.001
Magnesium *(1.9–2.7 mg/dL)*	on admission (159)	2 (1.8–2.2)	2.2 (1.9–2.6)	− 0.30 - -2.9e-5	0.014
	48–96 h (164)	2.2 (1.8–2.3)	2.4 (2.1–2.7)	− 0.4 - -0.2	< 0.001
BUN *(7–25 mg/dL)*	on admission (285)	22 (14–38)	33 (19–54)	− 14.0 - -5.0	< 0.001
	48–96 h (235)	20 (14–40)	38 (23–67)	− 22.0 - -10	< 0.001
Serum creatinine *(0.7–1.3 mg/dL)*	on admission (286)	1.3 (1.0–2.4)	1.7 (1.2–2.6)	− 0.5 - -0.1	0.008
	48–96 h (237)	1.2 (0.8–2.3)	1.6 (1.1–3.1)	−0.6 - -0.1	0.003
Glucose – random *(70–99 mg/dL)*	on admission (286)	128 (104–184)	182 (129–275)	− 61.0 - -23.0	< 0.001
	48–96 h (240)	103 (84–140)	163 (119–269)	− 72.9 - -35.9	< 0.001
AST *(13–39* ***μ****/L)*	on admission (284)	40 (26–65)	52 (38–83)	− 19.0 - -5.0	< 0.001
	48–96 h (224)	49 (28–66)	64 (37.7–106.2)	− 29.0 - -8.0	< 0.001
ALT *(7–52* ***μ****/L)*	on admission (284)	24 (16–38)	29 (19–44)	− 7.0 - 0.1	0.11
	48–96 h (224)	28 (17–52)	34 (22–57)	− 10.0 - 2.0	0.22
Alk Phosphatase *(34–104 U/L)*	on admission (284)	64 (49–78)	66 (54–96)	− 15.0 - -1.0	0.02
	48–96 h (223)	59 (46–78)	75 (52–111)	− 27.0 - -7.0	< 0.001
Bilirubin *(0.3–1 mg/dL)*	on admission (280)	0.5 (0.4–0.8)	0.6 (0.5–0.8)	− 0.1 - 5.0e-5	0.28
	48–96 h (219)	0.5 (0.4–0.8)	0.7 (0.5–.9)	− 0.2 - -5.4e-5	0.002
Total protein *(6–8.3 g/dL)*	on admission (282)	7 (6.5–7.3)	6.7 (6.4–7.2)	−2.6e-5 - 0.3	0.12
	48–96 h (219)	6.2 (5.9–6.6)	6 (5.5–6.7)	− 6.0e-5 - 0.4	0.10
Albumin *(3.5–5.7 g/dL)*	on admission (283)	3.6 (3.2–4.0)	3.4 (3.1–3.6)	0.1–0.3	< 0.001
	48–96 h (223)	3.0 (2.7–3.2)	2.8 (2.5–3.0)	0.1–0.3	< 0.001
LDH *(14–271 U/L)*	on admission (201)	379 (280–500)	551 (411–743)	106.0–22,849	< 0.001
	48–96 h (82)	406 (278–553)	638 (444.5–867)	106.9–339.0	< 0.001
CRP (< 10 mg/L)	on admission (201)	117 (63–197)	180 (128–283)	−97.0 - -36.9	< 0.001
	48–96 h (85)	96 (41–185)	283 (188–338)	−200.0 - -88.9	< 0.001
Troponin I (<=0.15 ng/mL)	on admission (170)	0.03 (0.02–0.12)	0.08 (0.02–0.21)	3.6e-5 - 0.06	0.010
	48–96 h (61)	0.11 (0.02–0.26)	0.15 (0.06–0.40)	− 0.03 - 0.18	0.30
Ferritin (14–233 ng/mL)	on admission (190)	654.5 (303–1151)	955 (539.0–2114.6)	118.5–566.5	0.002
	48–96 h (95)	768.5 (439–1821)	1614.1 (499.7–2801.5)	− 37.3, − 1036.7	0.08
Procalcitonin (0–0.10 ng/mL)	on admission(172)	0.32 (0.10–0.96)	1.03 (0.36–3.78)	0.19–0.88	< 0.001
	48–96 h (69)	0.34 (0.25–2.47)	1.68 (0.41–7.35)	4.75e-5 - 2.77	0.049
D-dimer < 0.4 mcg/ml	on admission (50)	3.3 (1.3–5.2)	1.5 (0.5–5.2)	−1.02 - 2.6	0.39
	48–96 h (43)	0.5 (0.5–1.5)	3.0 (1.1–7.5)	− 4.5 - -0.2	< 0.001
*Coagulation Parameters*					
aPTT (25.4–38.6 s)	on admission (126)	29.9 (28.4–32.4)	29.0 (26.9–33.6)	−1.9 - 1.5	0.68
	48–96 h (44)	30.7 (28.0–36.2)	31.1 (27.9–39.0)	− 6.3 - 6.3	0.99
PT (10.8–13.7 s)	on admission (113)	13.0 (12.2–13.7)	13.5 (12.6–15.4)	5.1–1.3	0.04
	48–96 h(43)	13.1 (11.9–15.2)	17.2 (13.3–20.2)	4.94e-5 - 6.7	0.04
INR (1 U)	on admission (113)	1.1 (1.0–1.1)	1.1 (1.0–1.3)	− 7.12e-6 - 0.10	0.07
	48–96 h (41)	1.0 (1.0–1.2)	1.4 (1.1–1.6)	7.49e-6 - 0.50	0.02

### Outcomes

**Fig. 3:**
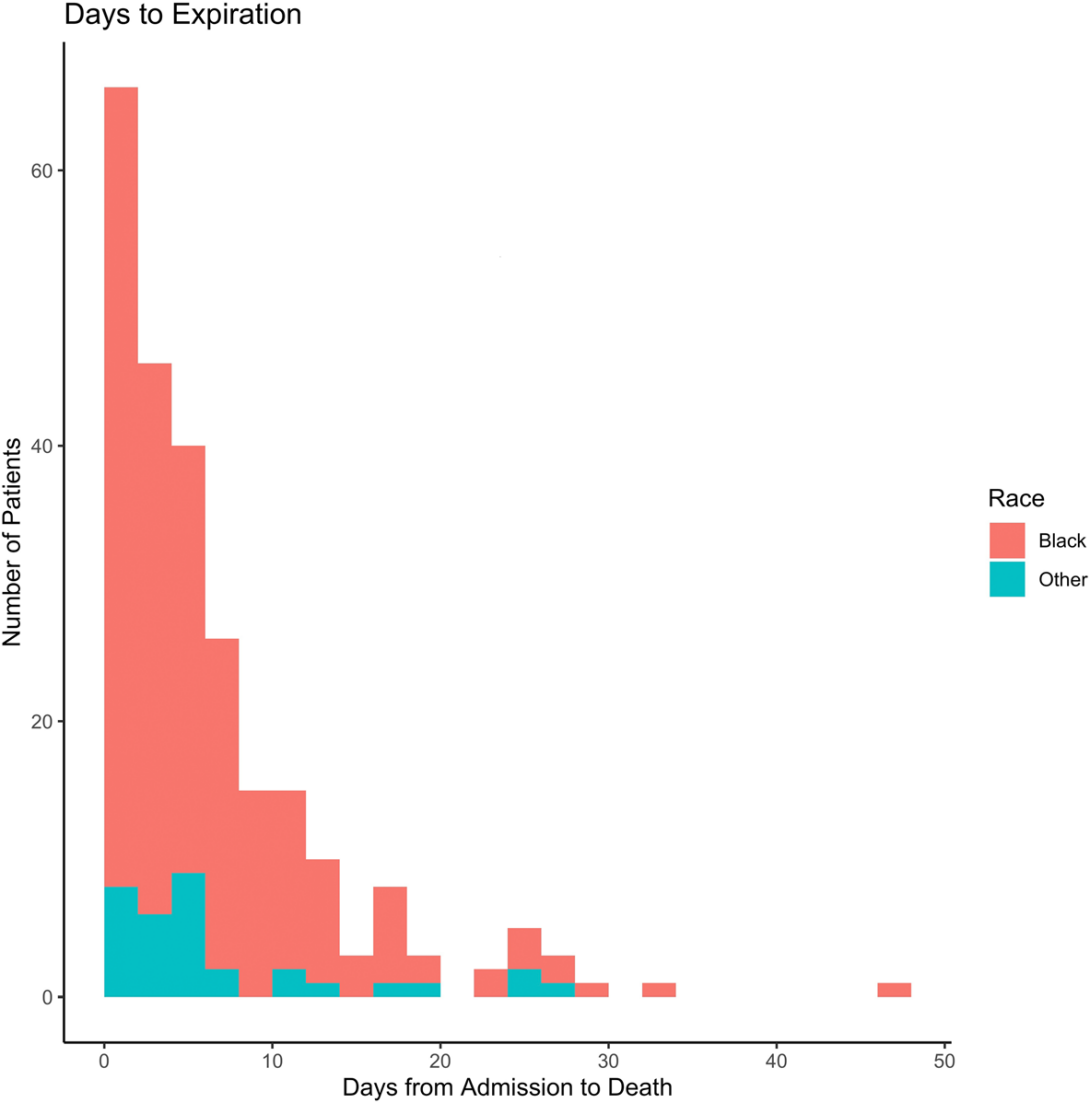
Days from admission to death of 255 consecutive inpatients. More than one third of patients (92/255) died within 3 days of admission for both Blacks (78/218) and Others (13/34)

Of the 529 hospitalized patients evaluated, 274 survived and 255 (48%) died by the end of the study. Of the 529 patients examined, 159 received invasive mechanical ventilation, of which 137 (86%) died. The remaining 370 patients who received supplemental oxygen therapy via non-invasive mode 123 (23%) died. This also included patients who self-declared “Do Not Intubate” (DNI), “Do not Resuscitate” (DNR) or came to the hospital in severe respiratory distress and died within the first few hours of admission. Of the patients who died, 36% (92/255) died in the first 3 days, which was similar for both Blacks (78/218) and Others (13/34) (Fig. 3). Patients who survived remained hospitalized from 1 to 37 (median: 6) days, and those who died were hospitalized from 0 to 47 (median: 5) days. Median time to death for mechanically ventilated patients was 5 days (range: 0–33) days, while for non-ventilated patients it was 4 (range: 0–47) days from admission.

## Discussion

This study documents the demographic, clinical features, and outcomes for patients admitted with COVID-19 at an urban hospital located in an underserved majority-Black neighborhood. We also identify indicators available to physicians at two early time points of evaluation to predict outcomes and develop management plans for appropriate levels of care.

The Black patient population in our study faces unique obstacles such as linguistic and cultural barriers to care and understudied comorbidities [13, 14]. Despite reports that African-Americans face significantly greater mortality from COVID-19, recent studies have examined the clinical outcomes in largely East-Asian or Caucasian cohorts [13]. Here, we present an analysis of 529 patients admitted with COVID-19, over a 52-day period at the height of the pandemic in New York City, and have either been discharged or died.

Older age at admission correlated with higher mortality rate, with the 60+ year age group most at risk, and was an independent risk factor for mortality. Males suffered significantly higher mortality than females, despite identical representation at admission. Recent reports of high plasma concentrations of ACE-2, a receptor for coronavirus, in men may account for higher mortality [15]. Our inpatient population had a mean BMI in the “obese” range, higher than the national average; this finding mirrors higher BMI amongst the Black population nationwide [16] However, BMI was not a predictor of survival; higher BMIs were more commonly seen amongst younger patients. Smoking was less prevalent in our patient population than the national average; 4% were current smokers and 15% had quit [17]. We found smoking to be unrelated to poor outcome.

The majority (88%) of our patients were Black. Race was not an independent prognostic factor for survival; higher mortality in our patient population can be attributed to a greater number and prevalence of comorbidities common amongst this group. Comorbidities were present in 98% of our patients, and the presence of any comorbidity was a strong predictor of mortality, as noted in other recent studies [18–20]. HT and DM were the two most prevalent preexisting conditions; prevalence of HT (79%) and DM (56%) was considerably higher than previously reported (up to 63 and 36%, respectively) [21–23]. In the multivariate analysis, coronary artery disease was strongly associated with adverse outcome (OR,2.38 CI, 1.11–5.50, *P* 0.03), followed by DM (OR, 1.22, CI, 0.81–1.84, *P* = 0. 35). A 2.5-fold increase in the risk of mortality from COVID-19 in hypertensive patients has been reported, however, this was not discernable in our patients [22]. Although past history of cancer, HT, autoimmune diseases, and immunosuppression were not independent predictors of mortality, the combined effect of these comorbidities on multiple organ systems and resultant dysregulation of the immune system likely increases susceptibility to COVID-19 [23, 24].

A notable finding in multivariate analysis was that patients with CKD who were dialyzed early in the course of treatment had better outcomes than those who did not (2%, OR, 0.27, CI, 0.04–1.11, *P* = 0.10). Although not statistically significant, we speculate that a larger number of patients with CKD on dialysis (currently *n* = 11) would allow for a definitive conclusion. These findings are notable considering patients with CKD had more comorbidities as compared to all other patients in the study. Early dialysis stands out as a potentially beneficial treatment option for patients with CKD. It is likely that dialysis removes inflammatory mediators, cytokines, and other effector molecules responsible for the end-organ damage. CKD and ESRD were more prevalent in our patient population (26%) than reported in other studies (between 3 to 8.5%), most likely due to complications from HT and DM [25].

We found laboratory data at admission vital for triaging patients to receive intensive care. CRP, LDH, and procalcitonin were significantly increased at both admission and at 48–96 h in non-survivors. Indicators of AKI, elevated levels of BUN, creatinine, glucose, and reduced levels of bicarbonate or albumin were significant predictors of adverse outcome at both initial and secondary time points. These findings correlate with reported tubular, endothelial, and glomerular capillary loop injury, likely the result of direct injury or systemic hypoxia [26]. Hypoproteinemia and hypoalbuminemia in non-survivors may result from renal insufficiency and suboptimal nutritional status in critically-ill patients, or could reflect stressed state [25]. As reported elsewhere, we found hyperglycemia to be a predictor of adverse outcome in COVID-19 patients, regardless of their history of diabetes [27]. Multivariate analysis of laboratory data was not performed due to sample size limitations.

Peripheral blood analysis showed that a high median NLR at admission and at 48–96 h was an independent predictor of adverse outcome in COVID-19 patients, as had been reported in other studies [28]. The presence of COVID-19 associated coagulopathy (CAC), a condition characterized by elevation in fibrinogen and D-dimer levels, high PT, relatively normal aPTT, and mild thrombocytopenia without evidence of microangiopathy, was confirmed in our study [29]. The mechanisms underlying CAC remain poorly understood, but it can possibly result from activation of extrinsic coagulation pathway, leading to excess consumption of Factor-VII following endothelial cell infection by the virus [30, 31] Elevated D-dimer levels at the second evaluation time point were associated with higher mortality, likely reflecting coagulation activation from sepsis, “cytokine-storm”, or impending organ failure.

By the end of our study, 48% of the inpatients had died, including 86% who received invasive mechanical ventilation. Reported mortality rates from other retrospective cohort studies ranged from 21% (New York metropolitan area) to 26% (Lombardy region, Italy) and 33% (UK) [4, 6, 32]. Relative to other studies, the mortality rate among our patients was elevated, which we believe is due to the largely poor and disadvantaged neighborhood where our hospital is located. Race was not found to be an independent predictor of mortality. Patients from similar underprivileged communities tend to present at an advanced stage of the disease leading to increased morbidity and mortality [33]. Rate ratios of hospital admission and mortality in US patients show a 4.7 and 2.1 times higher prevalence among Blacks as compared to Whites [34].

Our patients from a minority and underserved population had an unusually high burden of co-morbidities some of which proved to be independent predictors of the observed in-hospital high mortality; 1/3 of the patients died within the first 3 days of admission. We found some of the early laboratory data, together with demographics and co-morbidities, pivotal in predicting the clinical course of COVID-19. Early institution of dialysis in patients with chronic renal insufficiency reduced mortality significantly.

Our study has limitations. It examined a predominantly Black patient cohort, which makes comparisons to other races and ethnicities difficult to quantify. This study was carried out on patients admitted at the height of the pandemic in New York City, admissions were restricted to the most seriously ill and hospital resources were under strain, which may have contributed to an increase in overall mortality rates. Initiation of dialysis during admission occurred at the discretion of treating physicians, and there may be unmeasured differences between patients started on dialysis and those not-started on dialysis that are not accounted for in this analysis. As knowledge and understanding of COVID-19 was developing during March and April, complete laboratory studies were not systematically ordered for all patients. The routine use of steroids and Remdesivir were not established yet during the time of this study and so these findings, particularly the mortality rate, should be taken in that context. BMI was not included in the multivariate regression model as BMI was available in only a subset of patients.

## Conclusions

In our predominantly Black cohort we have recorded an in-hospital mortality rate from COVID-19 which is significantly greater than that reported in other studies. While race was not an independent predictor of death, this population had a greater burden of comorbidities than the national average and the prevalence of these chronic comorbidities contributed to both disease severity and higher mortality. Our study identified that early escalation of care is important in patients from minority neighborhoods as one third of the admitted patients die within the first 3 days of admission. Laboratory indicators at admission are predictors of outcome and can be utilized by physicians to triage patients and monitor disease course Early institution of dialysis in patients with chronic renal insufficiency trended toward association with lower mortality.

## Supplementary Information

**Additional file 1: Suppl 1.** Multivariate logistic regression analysis of the demographic characteristics and comorbidities for mortality. Dialysis has been added as a covariate for patients with ESRD and CKD. The presented odds ratios have been adjusted for multiple testing. CKD, chronic kidney disease; COPD, chronic obstructive pulmonary disease; ESRD, end-stage renal disease.

## Data Availability

The datasets used and/or analyzed during the current study are available from the corresponding author on reasonable request.
