# Supplementary figures and images for: Higher comorbidities and early death in hospitalized African-American patients with Covid-19
Source: BMC Infect Dis. 2021 Jan 18;21:78. doi: 10.1186/s12879-021-05782-9 (PMC7812567; doi:10.1186/s12879-021-05782-9)

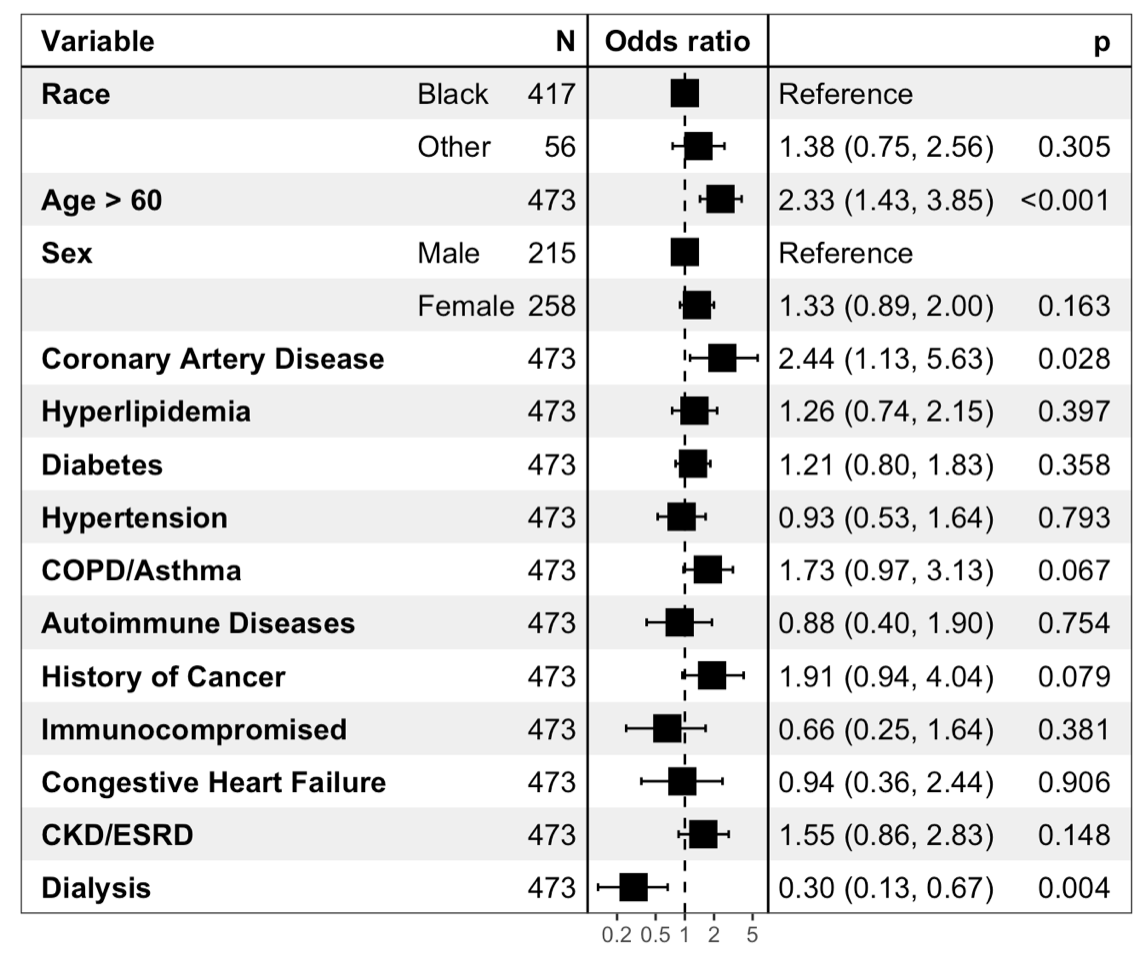

Supplement: Supplementary file 1 — Additional file 1: Suppl 1. Multivariate logistic regression analysis of the demographic characteristics and comorbidities for mortality. Dialysis has been added as a covariate for patients with ESRD and CKD. The presented odds ratios have been adjusted for multiple testing. CKD, chronic kidney disease; COPD, chronic obstructive pulmonary disease; ESRD, end-stage renal disease. [file 12879_2021_5782_MOESM1_ESM.png]
